# Biologic drugs in hidradenitis suppurativa: what does the GP have to know? A narrative review

**DOI:** 10.3389/fmed.2024.1403455

**Published:** 2024-07-08

**Authors:** Pedro Mendes-Bastos, Farida Benhadou, Marina Venturini, Alejandro Molina-Levya, Nicolas Thomas, Ivette Alarcon, Falk G. Bechara

**Affiliations:** ^1^Dermatology Centre, Hospital CUF Descobertas, Lisbon, Portugal; ^2^Department of Dermatology, Hôpitaux Universitaires de Bruxelles (H.U.B), Université Libre de Bruxelles (ULB), Brussels, Belgium; ^3^Dermatology Department, University of Brescia and ASST Spedali Civili Hospital, Brescia, Italy; ^4^Department of Dermatology, Hospital Virgen de las Nieves-Ibs.GRANADA, Granada, Spain; ^5^Novartis Pharma AG, Basel, Switzerland; ^6^Department of Dermatology, Venereology and Allergology, Ruhr-University, Bochum, Germany; ^7^ICH—International Center for Hidradenitis Suppurativa/Acne Inversa, Ruhr-University, Bochum, Germany

**Keywords:** hidradenitis suppurativa, biologic therapy, family medicine, diagnosis, screening, HS management, dermatology

## Abstract

Hidradenitis suppurativa (HS) is a chronic, inflammatory skin disease with a profound disease burden. In recent years, the advent of biologic therapies has improved the treatment landscape for patients with moderate to severe HS. In this new therapeutic era, the role of the general practitioner (GP) in HS treatment is becoming more important than ever. This review discusses how to recognize and diagnose HS by detailing common symptoms. HS can also present with multiple comorbidities. The GP’s role in screening for and treating these important comorbidities is pivotal. This review highlights the HS treatment landscape, with a specific focus on what the GP can recommend. The three approved biologics for treating HS include adalimumab, secukinumab and bimekizumab; the benefits and concerns of biologics in everyday clinical practice are detailed. In summary, this review serves as a HS management guide for GPs, with a particular focus on the biologic treatment landscape.

## Background

Hidradenitis suppurativa (HS) is a chronic, inflammatory, recurrent, painful skin disease, which is associated with a high disease burden, a substantial impact on patients’ quality of life (QoL) and multiple comorbidities (1–4). Despite being a relatively common disease, with a global estimated prevalence of approximately 1% (5), HS seems to be under-recognized and under-treated, and patients experience a significant delay in diagnosis of 7.2 to 10 years (6, 7).

In recent years, the disease paradigm has changed radically in patients with moderate and severe forms of HS with the advent of biologic drugs with immunomodulatory properties. For many years, the only biologic drug approved for the treatment of moderate to severe HS was adalimumab, a tumor necrosis factor-alpha (TNF-α) inhibitor (8, 9). More recently, secukinumab, an interleukin (IL)-17A inhibitor, and bimekizumab, an IL-17A and IL-17F inhibitor, have been approved for the treatment of moderate to severe HS (10–12). Furthermore, it is foreseeable that the biologic and small-molecule therapeutic arsenal will soon expand for HS treatment (9, 13, 14); hopefully this new era will increase the number of patients treated with biologics who will be jointly managed by dermatologists and general practitioners (GPs).

The rationale of this review is to empower GPs in managing HS, considering the anticipated increase in biologic treatment availability for HS. GPs form an integral part of HS management, and a multidisciplinary approach will ensure the optimization of clinical outcomes. This narrative review will serve as a guide for the management and treatment of HS for GPs, with a particular focus on biologic treatment. This will help optimize HS treatment in the era of biologics and provide practical solutions that may arise in routine consultation with these patients. The goals of this narrative review are to:

(I) Reduce the diagnostic delay of HS.
(II) Uniformize GP medical care for patients with HS.
(III) Facilitate GP referral to dermatologists.
(IV) Create a multidisciplinary network for HS management.

## What is HS and what does it look like?

HS is defined as a chronic, inflammatory, recurrent, debilitating skin disease of the terminal hair follicle, that usually presents after puberty, with painful, deep-seated, inflamed lesions in the apocrine gland-bearing area of the body, most commonly the axillary, inguinal, and anogenital regions (4). A common misconception is that HS is an infectious disease or a disease resulting from poor personal hygiene. Although it is not an infectious disease, dysbiosis is common in HS, and thus there is a possibility of superinfection and microbiome alterations as part of HS pathogenesis (15). The clinical diagnosis of HS is defined by three major diagnostic criteria (4):

- What do you see?
  o The presence of recurrent painful/purulent lesions/boils [inflammatory nodules, abscesses, and tunnels (fistula or sinus)] on the skin.
- Where do you see it?
  o The axillae, inframammary and intermammary folds, inguinal creases, perineal region, and buttocks.
- How often do you see it?
  o At least two lesions/boils within a period of 6 months.

**Figure 1:**
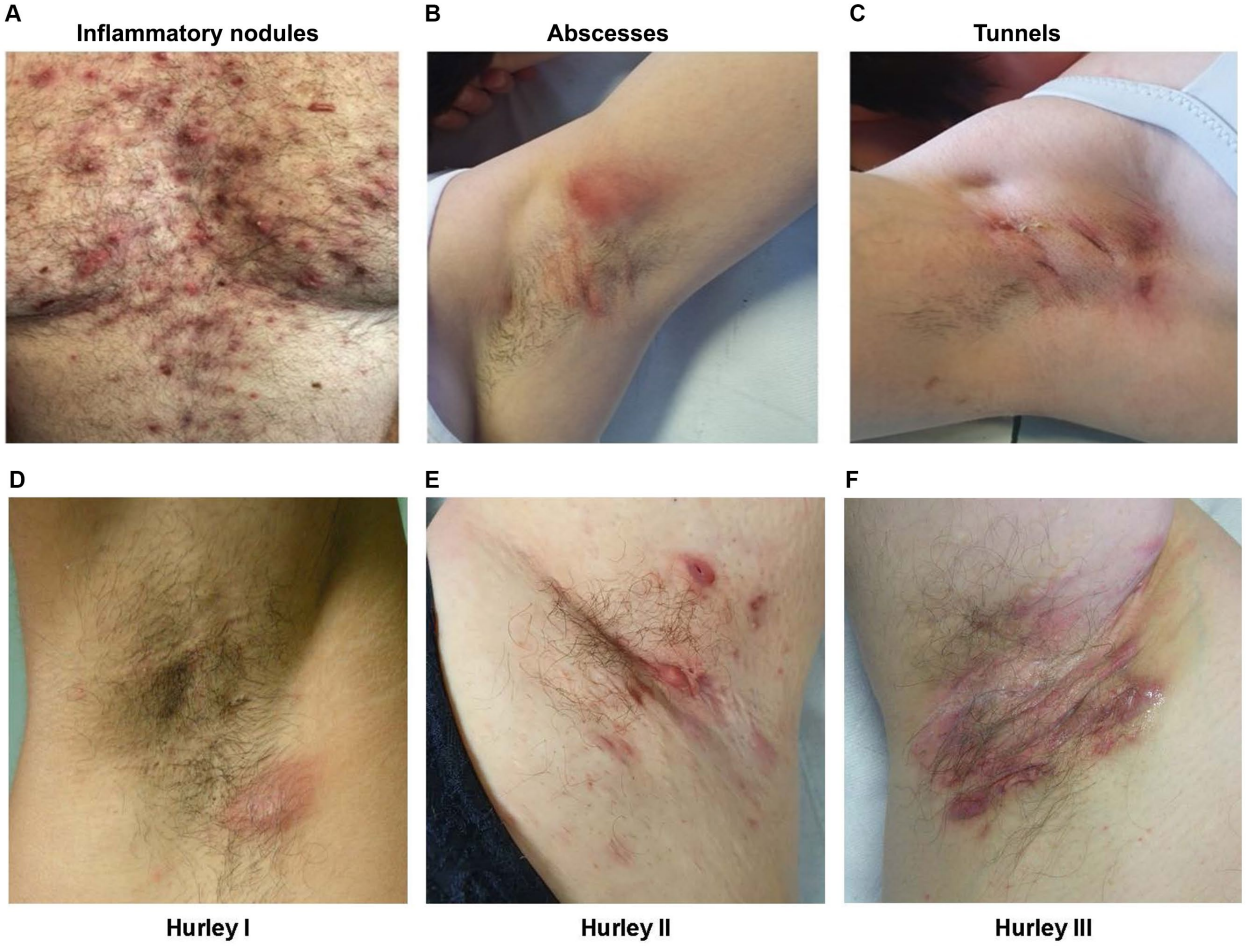
Clinical images of the most common HS lesions and the different severity stages of HS based on the Hurley staging system in the armpits of patients with HS. Lesions include **(A)** inflammatory nodules; **(B)** abscesses; and **(C)** tunnels, and Hurley staging includes **(D)** Hurley stage I; **(E)** Hurley stage II; and **(F)** Hurley stage III. Clinical images were provided by the authors with consent from the patients. HS, hidradenitis suppurativa.

Typical HS lesions include inflammatory nodules, abscesses, and tunnels (Figures 1A–C) (4, 16, 17), which are usually accompanied by discomfort, pruritus, and pain. Inflammatory nodules and abscesses are often erythematous and tender, with abscesses displaying fluctuance (16). Tunnels may open to the skin surface and form coalescing and interconnecting tracts within the dermis; drainage of malodorous material containing cellular debris, microbes, and pus and/or blood may be seen (16), which can cause emotional distress for the affected person (18). HS can also lead to scarring and changes in skin color and texture. The scarring resulting from HS can also be physically disfiguring.

The Hurley staging system is widely accepted and used for the classification of different HS disease severities (19). The Hurley staging system classifies HS into three stages based on structural damage, originally designed to help select surgical treatment for patients (Figures 1D–F) (19):

- Stage I: Single or multiple isolated abscesses without sinus tracts or scarring.
- Stage II: Recurrent abscesses with ≥1 sinus tracts and scarring, separated by normal skin.
- Stage III: Diffuse boils with multiple interconnected sinus tracts and no intervening normal skin.

However, the Hurley system is static and does not allow for a dynamic assessment of the extent of inflammation within each Hurley stage (19). More recently, the International Hidradenitis Suppurativa Severity Score System (IHS4) has been developed, which has a dynamic disease severity scoring system for HS (20). Calculating the IHS4 for a patient requires counting the number of nodules, abscesses, and draining tunnels and is calculated as (20):

$$\begin{array}{l} IHS4=\left(\mathrm{number}\;\mathrm{of}\;\mathrm{nodules}\times 1\right)+\left(\mathrm{number}\;\mathrm{of}\;\mathrm{abscesses}\times 2\right)\\ \;+\left(\mathrm{number}\;\mathrm{of}\;\mathrm{draining}\;\mathrm{tunnels}\times 4\right) \end{array}$$

Following the calculation, the total score categorizes patients based on their severity:

- ≤3 = mild HS.
- 4–10 = moderate HS.
- ≥11 = severe HS.

**Figure 2:**
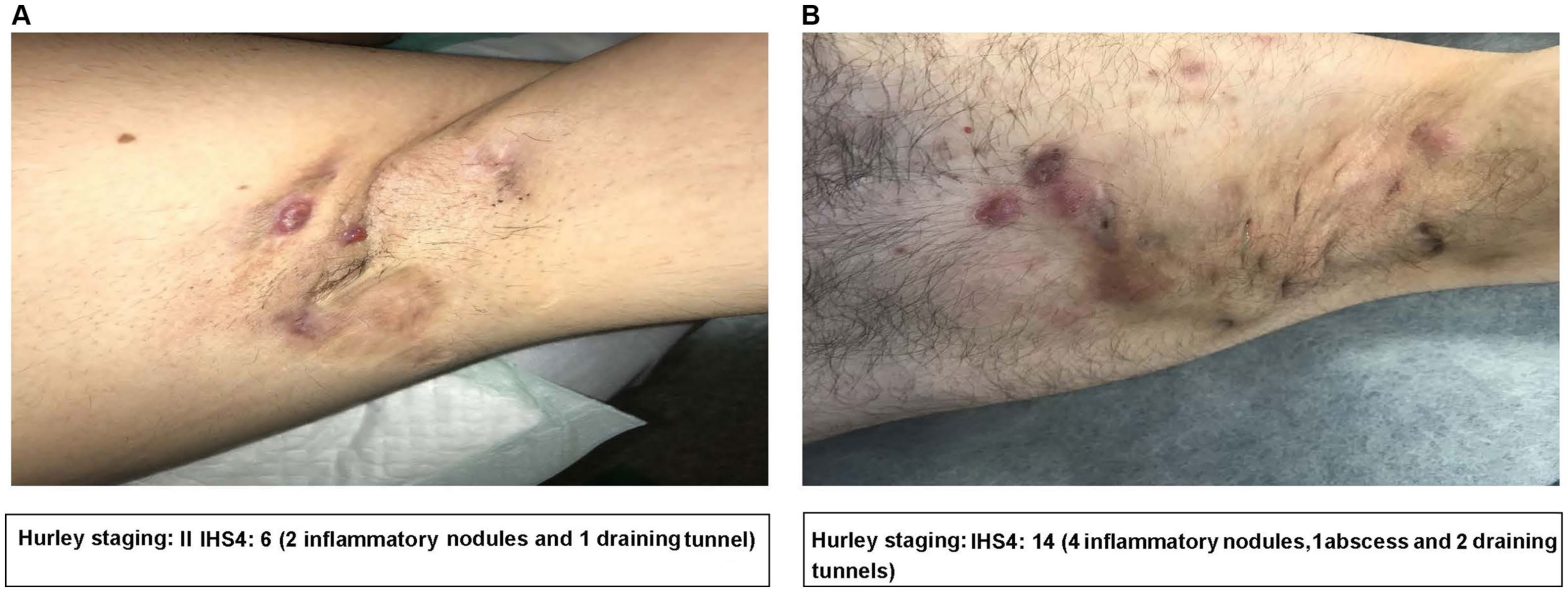
Clinical image examples of the Hurley staging system and IHS4 in practice. Figures detailing **(A)** a patient with Hurley stage II and an IHS4 of 6; and **(B)** a patient with Hurley stage II and an IHS4 of 14. Clinical images were provided by the authors with consent from the patients. IHS4, International Hidradenitis Suppurativa Severity Score System.

Although some patients may be classified as having the same Hurley staging, these patients may have different IHS4 staging. In Figures 2A,B, the patients both have Hurley stage II but have moderate (Figure 2A) and severe (Figure 2B) IHS4, highlighting the importance of a dynamic scoring classification system.

The complex and heterogeneous nature of HS has led to the observation and definition of different disease phenotypes, with the aim of improving disease prognostication and management (21). One such definition proposes that there are two main phenotypes of HS, based on lesion pattern; namely the follicular subtype and inflammatory subtype (22).

- Follicular subtype: This subtype is characterized by the presence of folliculitis and/or solid small pale papules on a background of comedones. The main active lesion of this subtype is the nodule. Abscesses are rare and tunnels are exceptional and non-coalescent. This subtype is more common in women.
- Inflammatory subtype: This subtype is characterized by the absence of folliculitis/comedones and by the presence of abscesses and thick fistular tracts that show confluence in poorly defined inflammatory and scarring plaques. This subtype is more frequent in men.

Patients with the inflammatory subtype have been shown to be more likely to progress to severe stages of the disease while those with the follicular subtype have demonstrated non-progressive disease. Additionally, some patients of the follicular phenotype can progress to an inflammatory phenotype, leading to a mixed phenotype (22).

## What comorbidities in patients with HS should GPs screen for?

HS is associated with multiple comorbidities that can contribute to impaired patient QoL (1, 3, 23). Both HS and the associated comorbidities can significantly increase the risk of mortality (24, 25); a study by Reddy et al. (25) reported that the adjusted 5-year mortality risk with HS was increased by 14% compared to controls, with the risk being further influenced by smoking and comorbidities.

Comorbidities including cardiovascular disease (CVD), metabolic syndrome, rheumatological disorders, and psychological disorders can be screened for and co-managed by GPs (23, 26). GPs can also advise patients on the importance of a healthy diet (27), referring to a nutritionist as necessary, and on smoking cessation strategies, as smoking is common in this population and is potentially linked to disease severity (23). The presence of these comorbidities associated with HS highlights the importance of a multidisciplinary treatment approach between GPs and dermatologists when treating these patients. The most common relevant comorbidities associated with HS that the GP should be aware of and screen for if a patient presents to a GP clinic are detailed in Table 1 (1, 23, 26, 28–30).

**Table 1:** The most common relevant comorbidities in patients with HS.

Comorbidity	Description
Cardiovascular disease	Hypertension: Obesity and tobacco use increase the risk of hypertension; the prevalence of hypertension in HS is between 7.8 and 56.3% MACE: Alongside metabolic disorders and lifestyle factors, chronic systemic inflammation in HS may support a link with cardiovascular disease. The adjusted incidence risk of MACE in patients with HS is 1.5 times that of controls
Psychological disorders	Depression: The prevalence of depression in HS is as high as 26.0% Generalized anxiety disorder: The prevalence of generalized anxiety disorder in HS is approximately 5.0% Suicidal ideation/Completed suicide: Patients with HS are reported to have a higher suicide rate than controls Substance use disorder: Due to disease-related pain, patients with HS may have an increased risk of substance abuse, with prevalence as high as 4.0%
Metabolic disorders	Obesity: Obesity is more common in HS than in controls and has a prevalence ranging from 5.9 to 73.1% Dyslipidemia: Dyslipidemia is more common in HS than in controls and has a prevalence ranging from 3.3 to 45.3% in patients with HS (adjusted odds: 1.4–4.1) Diabetes mellitus: Diabetes mellitus is more common in HS than in controls and has a prevalence ranging from 7.1 to 24.8% Metabolic syndrome: In conjunction with metabolic disorders, the chronic inflammatory state of HS may increase metabolic syndrome risk. It is more common in HS than in controls and has a prevalence ranging from 10.4 to 50.6%
Other disorders	IBD: Systematic reviews and meta-analyses have reported a significant link between HS and IBD (Crohn’s disease and ulcerative colitis). The prevalence of Crohn’s disease in HS is 0.2–2.0% and of ulcerative colitis is 0.3–1.3% Inflammatory arthritis: Spondyloarthritis and psoriatic arthritis are all more common in patients with HS than the general population Tobacco smoking: Self-reported smoking is higher in patients with HS than controls, ranging from 17.9 to 88.9%

Information has been obtained and adapted from Garg et al. (23). HS, hidradenitis suppurativa; IBD, inflammatory bowel disease; MACE, major adverse cardiovascular event.

### Screening for comorbidities

The ability of the GP to screen for comorbidities associated with HS is essential for the long-term management of patients. Garg et al. (31) have published comorbidity screening recommendations for primary care providers treating patients with HS.

CVD, obesity, and related conditions can be screened by conducting general CVD screening measures including anthropometry, blood pressure and fasted blood samples (lipid panel, glycosylated hemoglobin, blood glucose) (31). Lifestyle factors including diet, tobacco use, and physical activity levels can also be assessed (31). Psychological disorders can be screened for by using validated screening tools such as the Patient Health Questionnaire-2 and 9, Hospital Anxiety and Depression Scale, Columbia-suicide Severity Rating Scale, Generalized Anxiety Disorder 7-item scale, Opioid Risk Tool, and Alcohol Use Disorders Identification Test-C Questionnaire (31). Inflammatory bowel disease (IBD) and inflammatory arthritis can be initially screened by anamnesis and clinical examination; complementary tests such as a colonoscopy and peripheral and axial joint imaging, respectively, may be offered in cases of clinical suspicion (31).

## How is HS treated and what can the GP do?

**Figure 3:**
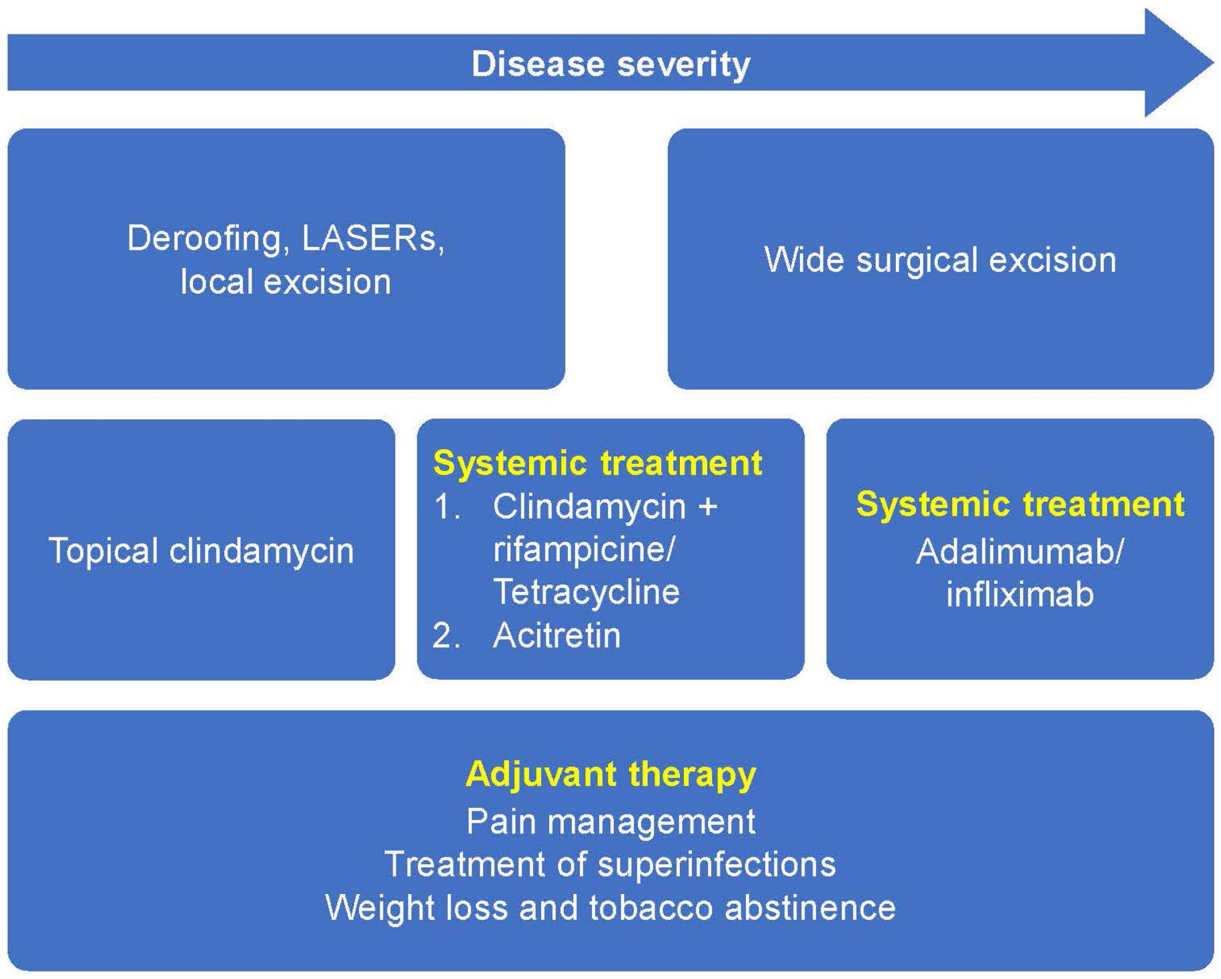
Current European guidelines for the treatment of HS based on disease severity. Reprinted with permission from Wiley (19), © 2015 European Academy of Dermatology and Venereology.

Presently, the standard treatment for HS includes a combination of medical and surgical treatments (19, 32). Current treatment guidelines recommend an escalating order of therapy, and the choice of treatment will depend on patients’ disease severity, disease features or phenotypes, and disease history (Figure 3) (19, 33).

In patients with mild forms of HS with discrete and sporadic lesions, management may be based on lesion-directed treatments. These range from medical treatments such as the use of topical antibiotics including clindamycin, high-dosage zinc, and corticosteroids, or minor surgical treatments such as incision and drainage or deroofing (19, 34). Laser hair removal may also be beneficial for mild forms of HS and is something that GPs can refer patients for, even though further studies are required to confirm it as a standard treatment for HS (19, 35).

In patients with moderate and severe forms of HS, in addition to treatments for recurrent acute lesions, a long-term, anti-inflammatory treatment is likely necessary to prevent the appearance of additional lesions and to favor the remission of existing lesions to improve QoL and prevent disease progression (19).

Adjuvant therapy can also be offered to patients in the form of general measures such as pain management, treatment of superinfections, weight loss, and smoking cessation (Figure 3) (19). Although there is a lack of data to show improvement of HS lesions after weight loss or smoking cessation, descriptive studies have shown a positive correlation between disease severity, body mass index (BMI), and cessation of tobacco smoking (19, 36). It is generally accepted that these measures should be encouraged in patients with HS who are overweight, obese or who smoke. For patients with HS who are obese, bariatric surgery associated weight loss may lead to HS improvement; however, severe malnutrition, a possible complication which can worsen or lead to new-onset HS post-bariatric surgery must be avoided (37).

### Defining a HS flare

Although flaring is a dominant manifestation of the disease, an accepted definition of a HS flare is not available, hindering its treatment (38). A HS flare has multiple definitions, with most definitions underpinning the term “exacerbation of symptoms.” A 2022 study reported that, following a Delphi consensus process, the definition of a flare was “a new or substantial worsening of clinical signs or symptoms.” (39).

### Lesion management

The choice of lesion directed treatment/acute lesion management (topical, intralesional, surgical) versus systemic medication/chronic lesion management should be based on a comprehensive evaluation of the patient, their personal preferences, and the clinical situation. Both approaches can be combined. Acute lesion management can be performed in discrete lesions chosen by the patient because they are the most symptomatic, or by their healthcare provider because they have risk of progression or complication. Systemic treatment can be recommended in widespread disease (affecting multiple body areas) or in patients with a single area with large involvement and significant inflammation.

Acute lesions can be managed through a combination of medical and surgical treatments including the following:

- Local topical treatments such as resorcinol (a keratolytic/peeling agent) for nodules and abscesses and clindamycin (an antibiotic with anti-inflammatory properties) for pustules (40, 41).
- Intralesional corticosteroids such as triamcinolone acetonide 5–10 mg/mL are advocated for the rapid reduction of inflammation associated with acute flares and for the management of nodules, abscesses and sinus tracts (19).
- Systemic corticosteroids (e.g., 0.5–0.7 mg/kg oral prednisolone) used in the short-term that are rapidly tapered may help reduce inflammation associated with flares (19).
- Systemic antibiotics are also widely used for flares (see below).
- For the management of acute pain, topical analgesics including topical lidocaine, oral acetaminophen, and oral non-steroidal anti-inflammatory drugs (NSAIDs) are the preferred treatment choice. Opiates can be considered for those resistant to other analgesics (42).
- Deroofing, to remove the “roof” of an abscess or inflammatory tunnel, appears to be effective for the treatment of acute lesions and is the preferred surgical intervention for individual lesions and sinus tracts due to its tissue-sparing nature and ability to be performed with local anesthesia (42–44).
- Incision and drainage provides acute relief but recurrence rates approach 100%. It is recommended only for acute abscesses for pain relief (42).
- For extensive Hurley Stage III HS, wide local surgery or carbon dioxide laser excision may be necessary to achieve disease control (44).

The initial management strategy for patients who present with chronic HS lesions is the administration of antibiotics including doxycycline 100 mg twice daily, tetracycline 500 mg twice daily, and lymecycline 300 mg twice daily (these doses were administered in a prospective study in HS patients, with a mean duration of treatment of 4.3 months) (45), or combinations including rifampicin 300 mg twice daily plus clindamycin 300 mg twice daily, for 10 to 12 weeks (19, 46). Hormonal therapies including anti-androgenic drugs such as spironolactone 100 mg to 150 mg daily, or the anti-diabetic drug metformin 500 mg 2–3 times daily have been shown to improve HS and should be considered in females as adjunctive agents for more severe disease (41). However, when there is symptom recurrence or a lack of disease control, it is necessary that patients are referred to a dermatologist to optimize care. The dermatologist can thereafter coordinate an individualized, multi-disciplinary approach on a patient-by-patient basis. Depending on the lesion, anatomical location, extent of scarring, access to general anesthesia, and the skills of the dermatologist, the patient may be managed solely in a dermatology center, or it may be necessary to involve other specialists experienced in HS, such as a general surgeon, colorectal surgeon, plastic surgeon, urologist, gynecologist, and so on. It can be a challenge, however, to create multidisciplinary teams familiarized with HS, and good communication between the dermatologist and other team members is crucial. The treatment plan is frequently dynamic and will be adapted according to the disease course and the response to different treatments.

In patients who have poor inflammatory control with antibiotics, the next step may involve the initiation of biologic therapies; adalimumab, secukinumab, and bimekizumab are the only biologics approved for treatment, and the choice of biologic will be specific to the patient’s history (9–12). The general recommended dose of adalimumab for adult patients with HS is 160 mg on day 1 (four 40 mg injections in 1 day or two 40-mg injections per day for 2 days), 80 mg 2 weeks later (two 40 mg injections in 1 day), and 40 mg injections every week or 80 mg (two 40 mg injections in 1 day) every other week thereafter as maintenance doses (47). The general recommended dose of secukinumab for adult patients with HS is 300 mg subcutaneous injection with initial dosing at weeks 0, 1, 2, 3, and 4, followed by every 4 weeks maintenance dosing. Based on clinical response, the maintenance dose can be up titrated to 300 mg every 2 weeks (48). The general recommended dose of bimekizumab for adult patients with HS is 320 mg (given as 2 subcutaneous injections of 160 mg each) every 2 weeks up to week 16 and every 4 weeks thereafter (49). Depending on the individual characteristics of each region, other treatments can be considered before or in combination with biologic treatment, such as oral acitretin (50, 51). Apart from the three mentioned biologic therapies, all other therapies are off-label in HS.

### Treating HS in children and adolescents

Pediatricians and GPs play a vital role in the early management and timely referral to dermatology clinics when managing children and adolescents with HS; early referral prevents disease progression and may improve medical outcomes. Although HS typically develops in adolescents after puberty, it can still present in children. However, data relating to the prevalence of pediatric HS are unclear (52). A 2018 report investigating the prevalence of HS in children and adolescents in the United States reported a prevalence of 0.028%, with 96.8% of cases in persons aged ≥10 years, with HS being more common in females than in males with a ratio of 3.8:1 (53). Given this, the treatment of children and adolescents with HS is challenging as there are limited data on the efficacy and safety of therapies, especially systemic treatments (52). From the available knowledge in the area, following a HS diagnosis in a pediatric patient, off-label treatment (except for adalimumab) may proceed as follows in conjunction with a dermatology referral (52, 54):

- Mild HS: Initiate treatment with topical antibiotics and non-pharmacological options including warm compresses and dilute bleach baths. The option of laser hair removal may also be useful for early cases of HS.
- Moderate to severe HS: Consider using oral antibiotics such as clindamycin for moderate disease in the first instance. Acetaminophen can be used for pain management. Females with pre-menstrual flares or polycystic ovary syndrome may benefit from treatment with metformin and hormonal modulators including spironolactone and oral contraceptive pills. The use of adalimumab can also be considered for patients aged ≥12 years and weighing ≥30 kg.

Along with medical treatments, particular attention should be paid to address the psychological factors associated with HS in children and adolescents by regularly monitoring patients’ mental health, as the disease can have devastating effects on mental and emotional well-being (54). Lifestyle modifications are important for children and adolescents and include weight loss, smoking cessation and the reduction of friction at intertriginous sites (54); these modifications will help HS management as well as help in the management of comorbidities.

## Why are biologics used in HS?

### Pathophysiology of HS

**Figure 4:**
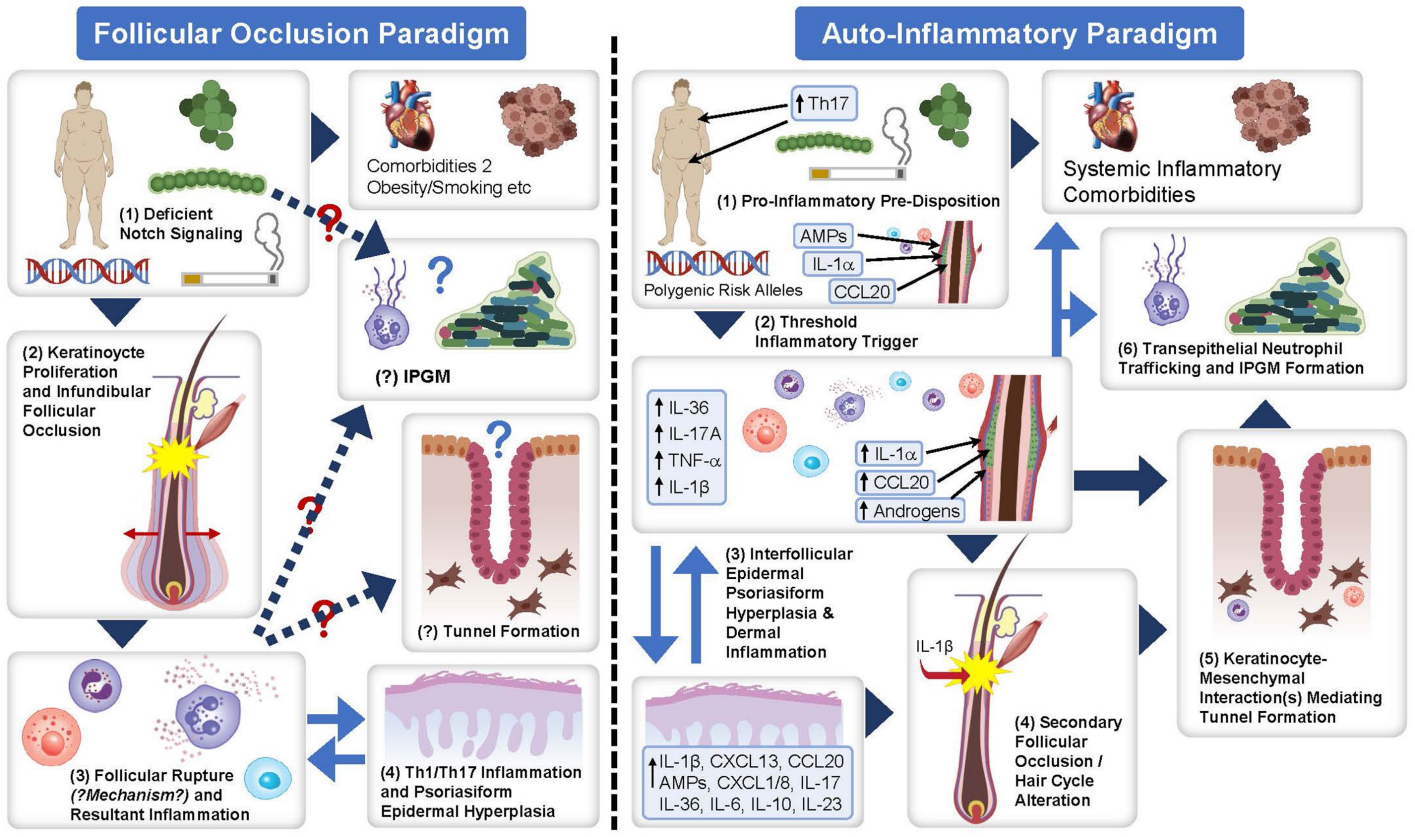
The follicular occlusion paradigm and auto-inflammatory paradigm of the pathophysiology of HS. Flow diagram detailing the follicular occlusion paradigm and auto-inflammatory paradigm for the proposed pathophysiology of HS. Figure obtained from Frew (55) under the CC BY NC ND license. AMP, adenosine monophosphate; CCL/CXCL, chemokine ligand; HS, hidradenitis suppurativa; IL, interleukin; IPGM, infiltrative proliferative gelatinous mass; Th, T-helper; TNF-α, tumor necrosis factor-alpha.

Understanding the pathophysiology of HS is imperative to understand the development and potential effectiveness of biologic therapies for HS treatment. The pathophysiology of HS is complex and not fully understood. However, histologic and molecular evidence supports the concept of inflammation as the primary driver of disease activity in HS, with immunologic, genetic, environmental and lifestyle factors contributing to disease development (Figure 4). A report by Frew (55) presents two different paradigms of HS pathophysiology: the follicular occlusion paradigm and auto-inflammatory paradigm. Although one paradigm cannot be displaced by the other, there is growing consensus that inflammation is the primary driver of HS pathophysiology (55). Briefly, the autoinflammatory paradigm highlights inflammation as the primary HS disease driver, with subclinical inflammation developing due to disparate contributing factors on a background of topographic predisposition (55). Dermal inflammatory infiltrates consequently drive secondary follicular occlusion, which can result in tunnel formation (55). This occurs because of keratinocyte-mesenchymal interactions that mimic outer-root sheath keratinocyte downgrowth in follicular development in early anagen (55). Chemokine gradients in epithelialized tunnels then drive neutrophil trafficking to the lumen and the formation of the infiltrative proliferative gelatinous mass leading to symptoms associated with HS (55). Many immune cells are involved in the pathogenesis of HS, including neutrophils, macrophages, T cells, and B cells, among others (55).

### Biologics and how they work

Owing to the prominent role of the inflammatory system in the pathogenesis of HS, the blockade of many immune cells has been targeted to treat HS, with successful trials reinforcing various immune cells’ role in HS pathogenesis (13). Several biologic therapies have been developed which block these immune cells, mainly cytokines (TNF-α, IL-17, IL-1, IL-23, IL-36), Janus kinases, and chemokines (13). Currently, adalimumab, secukinumab and bimekizumab are the only biologics that are approved for the treatment of moderate to severe HS (9–12).

### TNF-α

The blockade of TNF-α is a prominent pathway that is targeted (13). Adalimumab, a monoclonal immunoglobulin G1 (IgG1) antibody against TNF-α, is currently the only approved TNF-α biologic for the treatment of moderate to severe HS (9). In phase 3 trials of adalimumab (PIONEER I and II), the proportion of patients achieving HS Clinical Response (HiSCR) at week 12 (primary endpoint) was significantly higher with adalimumab administered weekly compared to placebo (41.8% vs. 26.0% in PIONEER I; 58.9% vs. 27.6% in PIONEER II, respectively) (8). Adalimumab biosimilars are available; however, studies investigating the switch from the originator to the biosimilar in patients with HS are lacking. A recent retrospective study found no significant differences in terms of clinical response following the switch (56). Other anti-TNF-α therapies include infliximab (off-label use), a chimeric monoclonal IgG1 antibody against TNF-α.

### IL-17

IL-17 was selected as a target for pharmacological agents due to its central role in HS pathophysiology (13). The isoforms IL-17A, IL-17C, and IL-17F have all been identified in the lesions of HS skin (13). The most prominent drugs that target IL-17 include secukinumab (IL-17A inhibitor), a human IgG1κ monoclonal antibody that was the first licensed IL-17A inhibitor for use in the EU and the US, and bimekizumab (IL-17A and IL-17F inhibitor), a humanized monoclonal antibody, currently licensed for use in the EU (10–13).

Phase 3 trials utilizing secukinumab (the SUNSHINE and SUNRISE trials) have reported that the proportion of patients achieving HiSCR at week 16 (primary endpoint) was significantly higher with secukinumab every 2 weeks versus placebo (45% of 181 patients vs. 34% of 180 patients respectively in the SUNSHINE trial; 42% of 180 patients vs. 31% of 183 patients respectively in the SUNRISE trial) and with secukinumab every 4 weeks versus placebo in the SUNRISE trial (46% of 180 patients vs. 31% of 183 patients respectively), with efficacy sustained to 52 weeks of treatment (57).

Phase 3 trials utilizing bimekizumab (BE HEARD I and BE HEARD II) have reported that the “proportion of patients achieving HiSCR” to be consistent with Secukinumab and adalimumab at week 16 (primary endpoint) was significantly higher with bimekizumab every 2 weeks versus placebo (48% of 289 patients vs. 29% of 72 patients in the BE HEARD I trial; 52% of 291 patients vs. 32% of 74 patients in the BE HEARD II trial) and with bimekizumab every 4 weeks versus placebo in the BE HEARD II trial (54% of 144 patients vs. 32% of 74 patients), with efficacy sustained to 48 weeks of treatment (58).

In addition, in the real-life setting, there is some evidence to support the role of this drug class in patients with HS refractory to anti–TNF-α therapy (59).

### IL-1

Targeting IL-1 may be beneficial due to the activation of IL-1 in the pathogenesis of HS (13). Anakinra, a recombinant human antagonist of IL-1 (blocking both IL-1α and IL-1β) and lutikizumab (a dual-variable-domain IL 1α/1β antagonist) have shown some promise for the treatment of HS (13, 60). The clinical trial program for lutikizumab is progressing to phase 3, following recently reported positive phase 2 trial results (60).

### Other immune cells

Janus kinases are another class of immune cells that act as signal transducers of activated cytokines, thus blocking these cells blocks subsequent cytokine activation (13). Examples include tofacitinib, upadacitinib, and ruxolitinib (13). Other immune cell targets include chemokines, specifically CXC chemokines, S100A proteins, and IL-1 receptor-associated kinases (13).

## What concerns should GPs have during biologic usage?

### Vaccination for patients while using biologics

Currently, there are no specific guidelines or recommendations on vaccination in patients with HS using biologics. However, extrapolations can be made by examining guidance in other immune-mediated diseases treated with biologics. There are recommendations for vaccination in patients with both psoriasis and atopic dermatitis who are being treated with biologics (61–63), with a general consensus being that attenuated live vaccines should be avoided while using biologic agents, but non-live vaccines can be administered safely without affecting vaccine-induced antibody production (61, 62).

If a live vaccine is indicated, it should be administered 14 to 30 days prior to therapy initiation or ≥3 months after cessation of biologic therapy (63). Administration of the coronavirus disease 2019 (COVID-19) vaccination in patients being treated with biologics is considered safe, with patients recommended to take booster doses in a timely manner (63). Moreover, the administration of the varicella zoster virus vaccine in patients aged >50 years while on systemic therapies has been recommended as safe by the National Psoriasis Foundation, with treatment of patients <50 years being considered on a case-by-case basis (64).

### Risk of tuberculosis and other infections

As biologics are immunomodulatory agents, there is an inherent increased risk of infection with their use. Noteworthy, rare infections include tuberculosis (TB), with common infections including candidiasis and respiratory tract infections. There is an increased risk of reactivating latent TB infection with the use of TNF inhibitors due to the central role TNF has in maintaining TB in its latent phase due to granuloma formation (65). TNF inhibitors mainly do this by disrupting the granuloma formation process which usually compartmentalizes *Mycobacterium tuberculosis* during latent TB infection (65). Thus, it is advisable to screen patients for active and latent TB infection before commencing anti-TNF treatments. Medical practitioners should refer to and act accordingly with local country guidelines regarding TB screening and subsequent treatment.

There is an increased risk of various forms of candidiasis (oropharyngeal, esophageal, and cutaneous) with IL-17 inhibitors due to the involvement of IL-17 in anti-*Candida* host defenses (66). Therefore, patients should be closely monitored for these infections. The use of antifungal therapies in parallel with IL-17 inhibitors may be necessary for symptomatic patients. Additionally, antifungal prophylaxis should be considered for patients with recurrent or chronic candidiasis (66), although prophylactic antifungal therapy is not commonly used in HS clinical practice.

### Pregnancy and biologic treatment

Overall, caution should be taken during treatment with biologics throughout pregnancy, with more data needed for many biologic treatments. However, an individualized risk benefit discussion should be had with patients regarding treatment (67). Pregnancy in HS is a sensitive topic and should be approached with the patient with caution, as HS can lead to decreased fertility, and adverse reactions during pregnancy, as well as gestation triggering a clinical worsening of the disease (68). Therefore, biologic treatments in pregnancy should be discussed with patients of childbearing potential. There are three main considerations for biologic treatment in pregnancy including (1) before pregnancy (2), during pregnancy, and (3) while breast-feeding.

### Before pregnancy

There is mixed evidence for the use of biologics prior to pregnancy in terms of conception rates. In the psoriasis population, exposure to biologics during conception does not appear to have any adverse reactions, but more research in the field and in HS populations is warranted (69).

### During pregnancy

If the patient, dermatologist, GP and other members of the care team make a shared decision that the patient will remain on biologic therapy during pregnancy, TNF inhibitors are advised for use due to more data availability with these therapies during pregnancy (67), and/or the prescribing information of additional therapies should be consulted. Future studies should investigate the safety of secukinumab and bimekizumab as more recently approved therapies for patients with HS. It is advised that monoclonal antibody therapy is stopped during the third trimester of pregnancy to avoid placental transfer, as the placenta is most permeable to maternal IgG antibodies during this period (67, 70). If a fetus is exposed to biologic therapy during the third trimester, an infant’s vaccination scheme may need to be altered due to biologic circulation still occurring. The management of the vaccination scheme should be discussed between the patient, GP, dermatologist, and pediatrician to come to a shared decision.

### While breast-feeding

The safety of using biologics while breast feeding is unclear. However, current data suggest that there are no safety risks for the infant, as there are minimal amounts of the medications excreted through breast milk. Nevertheless, more data are warranted (67, 70).

### Managing surgery while using biologics

Surgery is an integral component for the management of HS and can alleviate pain and symptoms associated with HS (19). A study investigating surgery in parallel with adalimumab treatment in HS reported that adalimumab was efficacious in conjunction with wide-excision surgery, with no increased risk of postoperative wound infection, indicating no requirements for interrupting biologic treatment prior to surgery (71). Furthermore, the safety profile in this study was similar to that in studies using adalimumab alone, but more studies are needed to strengthen the evidence (71). Moreover, other biologics and surgical procedures have not yet been investigated which limits the evidence on the efficacy and safety of combining different biologics with different surgical procedure types. By extrapolating recommendations from psoriasis cohorts, it is likely that biologic therapies can be continued during minor surgeries, but for moderate- to high-risk surgeries, a case-by-case approach should be taken, which considers patient comorbidities, clinical history, and the benefit-risk of continuing biologics (72).

## Summary and conclusions

This narrative review aims to serve as a management guide for GPs, with a particular focus on biologic treatment, with the hope to bridge the gap between primary care and specialist dermatology care for HS. HS is a chronic, underrecognized, inflammatory skin disease associated with a high disease burden and comorbidity. With the advent and development of new biologic treatment options, it will be imperative to ensure close collaboration between GPs and dermatologists to ensure timely diagnosis and treatment, to manage comorbidities, and improve clinical outcomes for patients with this debilitating disease. HS is treated through an escalating order of medical and surgical treatments. Biologic therapies are becoming increasingly prominent and important for the management of HS. Caution and awareness of adverse events associated with biologics are advised, as well as a good understanding of vaccination, pregnancy, and surgery with biologic treatment for these patients.

## Author contributions

PM-B: Conceptualization, Methodology, Writing – original draft, Writing – review & editing. FaB: Conceptualization, Methodology, Writing – original draft, Writing – review & editing. MV: Conceptualization, Methodology, Writing – original draft, Writing – review & editing. AM-L: Conceptualization, Methodology, Writing – original draft, Writing – review & editing. NT: Conceptualization, Funding acquisition, Methodology, Resources, Supervision, Writing – original draft, Writing – review & editing. IA: Conceptualization, Funding acquisition, Methodology, Resources, Supervision, Writing – original draft, Writing – review & editing. FB: Conceptualization, Methodology, Writing – original draft, Writing – review & editing.
